# Effects of Allicin on Late Sodium Current Caused by ΔKPQ-SCN5A Mutation in HEK293 Cells

**DOI:** 10.3389/fphys.2021.636485

**Published:** 2021-03-29

**Authors:** Yating Chen, Yun Huang, Jing Bai, Chuanbin Liu, Shanshan Ma, Jiaxin Li, Xu Lu, Zihao Fu, Lihua Fang, Yang Li, Jiancheng Zhang

**Affiliations:** ^1^Department of Cardiology, Fujian Provincial Hospital, Provincial Clinical Medicine College of Fujian Medical University, Fuzhou, China; ^2^Department of Gerontology, Union Hospital, Tongji Medical College, Huazhong University of Science and Technology, Wuhan, China; ^3^Medical School of Chinese People’s Liberation Army (PLA), Beijing, China; ^4^Department of Cardiology, The Sixth Medical Center, Chinese PLA General Hospital, Beijing, China

**Keywords:** Allitridum, ΔKPQ-SCN5A mutation, HEK293 cells, late sodium current, LQT3

## Abstract

**Aim:**

The aim was to study the effect of Allitridum (Allicin) on the heterologous expression of the late sodium current on the ΔKPQ-SCN5A mutations in HEK293 cells, with a view to screening new drugs for the treatment of long QT syndrome type 3 (LQT3).

**Methods and Results:**

The ΔKPQ-SCN5A plasmid was transiently transferred into HEK293 cells by liposome technology and administered by extracellular perfusion, and the sodium current was recorded by whole-cell patch-clamp technology. Application of Allicin 30 μM reduced the late sodium current (*I*_*Na,L*_) of the Nav1.5 channel current encoded by ΔKPQ-SCN5A from 1.92 ± 0.12 to 0.65 ± 0.03 pA/pF (*P* < 0.01, *n* = 15), which resulted in the decrease of *I*_*Na,L*_/*I*_*Na,P*_ (from 0.94% ± 0.04% to 0.32% ± 0.02%). Furthermore, treatment with Allicin could move the steady-state inactivation of the channel to a more negative direction, resulting in an increase in channel inactivation at the same voltage, which reduced the increase in the window current and further increased the inactivation of the channel intermediate state. However, it had no effect on channel steady-state activation (SSA), inactivation mechanics, and recovery dynamics after inactivation. What’s more, the Nav1.5 channel protein levels of membrane in the ΔKPQ-SCN5A mutation were enhanced from 0.49% ± 0.04% to 0.76% ± 0.02% with the effect of 30 mM Allicin, close to 0.89% ± 0.02% of the WT.

**Conclusion:**

Allicin reduced the late sodium current of ΔKPQ-SCN5A, whose mechanism may be related to the increase of channel steady-state inactivation (SSI) and intermediate-state inactivation (ISI) by the drug, thus reducing the window current.

## Introduction

The congenital long QT syndrome type 3 (LQT3) is an autosomal hereditary arrhythmia that is clinically characterized by prolonged QT interval on a surface electrocardiogram (ECG), resulting in malignant arrhythmia with pleomorphic ventricular tachycardia and Torsade de Pointes (TdP), frequently synonymous with syncope and even sudden cardiac death (Bohnen et al., 2017; Garcia-Elias and Benito, 2018). LQT3 is the third most common of the 15 forms of the hereditary long QT syndrome (Schwartz et al., 2012) and demonstrates the largest proportion of lethal cases (Splawski et al., 2000). LQT3 diseases are associated with gain-of-function mutations in the Nav1.5 channel (Zimmer and Surber, 2008). However, the numerous LQT3 mutations contribute to distinct phenotypes of LQT3. Channel mutants, such as the ΔKPQ (Fredj et al., 2006), are characterized by a late Na^+^ current (*I*_*Na,L*_). The mutant channels show an increased influx of Na^+^ that increases the action potential duration (APD) and prolongs repolarization excessively (Chadda et al., 2017; Yu et al., 2018). Therefore, interfering with the incidence pathway and looking for new anti-LQT3 therapeutic medicines would be a development path in the future.

Allitridum (Allicin) is the main bioactive component of garlic in the genus *Allium* of Liliaceae with a potential therapeutic value in the prevention and treatment of cardiovascular diseases (Cui et al., 2020; Mocayar Maron et al., 2020). It was previously found that Allicin could shorten APD through the effect of the transient outward potassium current in ventricular myocytes (Cao et al., 2016) and the ultrarapid delays rectifier potassium current in atrial myocytes (Deng et al., 2011), which reduces the occurrence of arrhythmias. Nevertheless, it cannot assert that Allicin, relative to the *I*_*Na,L*_, would have similar effects on APD. The aim of this study was to describe the effects of Allicin on the *I*_*Na,L*_ carrying the ΔKPQ mutation with the extracellular direct perfusion method. We found that Allicin reduces the *I*_*Na,L*_ of the cardiac Na^+^ channel (ΔKPQ-SCN5A), whose mechanism may be associated with the dynamics of channel steady-state inactivation (SSI) and intermediate-state inactivation (ISI) by the drug, thus reducing the window current. These results provide an experimental basis for subsequent treatment of LQT3.

## Materials and Methods

### Medication

All chemicals were purchased from Sigma-Aldrich. The pure Allicin substance (Hebei Zhitong Biopharmaceutical Co., Ltd.) is an oily agent with a relative molecular mass of 162 g/mol that is colorless or yellowish. The stock solution should be prepared by dissolving Allicin in advance with dimethyl sulfoxide. Until use, it should be diluted with extracellular solution and then added to the extracellular solution to form a final concentration of 30 μM. In order to ensure the consistency and stability of the drug effect, the current was recorded after 5 min of high-impedance sealing and membrane breaking. In addition, a blank solution of the same volume as dimethyl sulfoxide was added to the extracellular solution, and it was found that there was no significant effect on the current.

### Cell Culture and Transfection

HEK293 cell line was cultured, and well-cultured HEK293 cells were observed under a microscope. The cells were grown in a Dulbecco’s modified Eagle’s medium [10% fetal bovine serum (Invitrogen), 2 mM L-glutamine] and stored at 37°C in a humidity-controlled incubator with 5% CO_2_. According to the instructions in the kit, Lipofectamine-2000 transfection reagent (Invitrogen) for transfection was used, and 0.2 μg green fluorescent protein (GFP) expression plasmid was co-transfected with the plasmid containing the target gene as a positive indicator of transfection, and 0.5 μg of the target plasmid pcDNA3.1-ΔKPQ-SCN5A of the corresponding volume required for transfection was added. Then, 72 h after transfection, the cells were transferred to 35 mm culture dish at a ratio of 1:10 and placed in the cell incubator for 4 h. The transfection was observed under a fluorescence microscope, and the positive cells were recorded by patch-clamp.

### Electrophysiological Recordings

The cells with clear edges, smooth surface, moderate size, spherical or polygonal, not attached to other cells, and with green fluorescence were selected for operation under the inverted fluorescence microscope. All recordings were carried out at room temperature (20–22°C). We used an Axon Multi-clamp 700B amplifier (Molecular Devices, United States), digidata 1440A acquisition interface (Molecular Devices, United States), and pClamp software (version 10.4; AXON Corporation, United States). The extracellular solutions contained (in mM): NaCl 130, CsCl 10, CaCl_2_ 2, MgCl_2_ 1.2, HEPES 10, and glucose 5, and the pH was adjusted to 7.2 with NaOH. The pipette solutions contained (in mM): K-aspartame acid 80, CsCl 60, MgATP 5, EGTA 10, GTP 0.1, and HEPES 10, and the pH was adjusted to 7.4 with CsOH. The GG-17 glass blank was drawn by pp-83 microelectrode puller (Narishige Corporation, Japan) to make an electrode with a tip diameter of 2–4 μm, and the pipette resistance ranged from 2.0 to 5.5 MΩ. After the electrode was put into the liquid, the potential was compensated and corrected to make the potential less than ±2 mV. The three-dimensional micromanipulator (Sutter Corporation, United States) was adjusted to make the electrode tip move to the cell surface for sealing, so that the resistance reached more than 1 GΩ to form gigaohm seal. Fast capacitance compensation was used to eliminate the capacitance error generated by the instrument. The pulse mode was used to break the cell membrane under negative pressure to form a whole-cell recording mode. When measuring the capacitance, the current was measured by the ramp stimulation of 0.4 V/s and calculated according to the equation *Cm*=*I*/(*d*_*V*_/*d*_*t*_) (*Cm* is the membrane capacitance, *I* is the current value, *d*_*V*_/*d*_*t*_ is the voltage slope). The whole-cell patch-clamp recording method was used to record the current under voltage clamp. In order to eliminate the error between the cells, the *I* value is expressed as current density (pA/pF). The signal is filtered by a fourth-order Bessel low-pass filter with a cut-off frequency of 1 kHz, and the sampling rate is 5 kHz. The series resistance was compensated by 90–95% to minimize the voltage clamp errors; the application instrument automatically performed about 85–90% slow capacitance compensation to eliminate the effect of charge and discharge caused by cell membrane capacitance. In all experiments, the cells were stabilized for 2–3 min after the cell membrane was broken, then the data before and 5 min after drug perfusion were recorded, and the data were recorded after 5 min of drug perfusion. In order to avoid the effect of rundown of the channel current on the experimental results, the experiment was completed within 20 min after cell membrane breaking (Dan et al., 2015; Zhang et al., 2015). The sampled data were stored for measurement and analysis of the electrophysiological characteristics of the channel.

### Current Recording Program and Data Analysis

*I*_*Na*_ recording: a depolarized pulses of −120 to −20 mV at a holding potential of −120 mV, with a step of 10 mV and a time duration of 50 ms in each pulse, was used to record *I*_*Na*_.

Steady-state activation (SSA) curves of *I*_*Na*_: a depolarized pulse of −120 to 20 mV, 500 ms was applied at a holding potential of −90 mV, with a step of 10 mV, to record the activity. The current is standardized, with the stimulus pulse at each voltage as the horizontal axis and the standardized tail current as the longitudinal axis. Using the Boltzmann equation (*G*/*G*_*max*_ = 1/{1 + exp [(*V*_1/2_ − *V*_*m*_)/*k*]}) to fit the curve, the half-activation voltage (*V*_1/2,act_) and the slope of the activation curve (*k*_*act*_) were calculated.

Steady-state inactivation curves of *I*_*Na*_: a fast pulse of −140 to −30 mV, 1,000 ms was applied at a holding potential of −90 mV, with a step of 10 mV, followed by a test pulse with a fixed depolarization to −30 mV, 50 ms to record *I*_*Na*_. Using the Boltzmann equation (*I*/*I*_*max*_ = 1/{1 + exp [(*V*_1/2_ − *V*_*m*_)/*k*]}) to fit the curve, the half-inactivation voltage (*V*_1/2,inact_) and the slope (kinact) of the curve are calculated (Clatot et al., 2017).

Intermediate-state inactivation curves of *I*_*Na*_: it was derived by a dual stimulation model, which was applied with −10 mV stimulation respectively at 1, 2, 5, 10, 25, 50, 100, 250, 500, 2,000, and 5,000 ms. Then the potential was restored to −100 mV, and a stimulation of −10 mV, 25 ms was given after an interval of 20 ms, before the current was recorded. The curve was fitted by monomial exponential formula, and the ISI time constant (*τ*) was calculated.

Closed-state inactivation curves (CSI) of *I*_*Na*_: it was derived by a dual stimulation model with a holding potential of −80 mV. −70 mV stimulations respectively at 1, 2, 4, 10, 20, 50, 100, 200, 400, 500, 1,000, and 2,000 ms were applied. Then stimulation of −10 mV, 25 ms was given before the current was recorded. It determined the availability of the channel during stimulation. Using the monomial exponential formula, *I*/*I*_*max*_ = *I*_0_ + A × exp (*t*/*τ*) was used to fit the curve and the CSI time constant (*τ*) was calculated (Chen et al., 2018).

### Confocal Imaging

After the transfected HEK293 cells were cultured for 36 h, they were rinsed with cold phosphate-buffered saline (PBS) twice and then were fixed with 4% polyformaldehyde for 15 min. After being rinsed with cold PBS again, the cells were treated with 0.3% Triton X-100 to permeabilize. After the above treatment, 10% normal goat serum was added to block non-specific sites. The cells were incubated with rabbit-anti-Nav1.5 primary antibody and GFP fluorescence secondary antibody, and then the appropriate amount of anti-fluorescence quenching sealing solution was fixed. The samples were examined using a Zeiss Ism 510 Meta fluorescence confocal imaging microscope, and the images were collected.

### Western Blotting

Western Blotting were performed on the proteins extracted from membranes of cultured cells. Protein samples were equally loaded on 8% sodium dodecyl sulfate (SDS) polyacrylamide gels and transferred onto nitrocellulose membranes. The membranes were blocked with 5% non-fat milk for 1 h at room temperature and then incubated with the specified primary antibody overnight at 4°C. After 1-h incubation with the required secondary antibody, the particular signals were revealed by the chemiluminescence detection reagent Western lightning plus-ECL. Primary antibodies used in this study included mouse anti-GAPDH monoclonal (1:1,000; Proteintech) and rabbit anti-Nav1.5 polyclonal (1:1,000; Abcam). Western Blotting data densitometric analysis was conducted using ImageJ software.

### Statistical Analysis

All the data were expressed as mean ± SD, and data analysis was done with pCLAMP 10.4 (Axon Instruments) and SPSS19.0 (Microcal Software). For multiple group comparisons, statistical significance was determined by ANOVA. With an ANOVA followed by a Student–Newman–Keuls (S–N–K) *post hoc* test, significance between any two groups was evaluated. *P* < 0.05 was considered statistically significant. The difference was statistically significant (*P* < 0.05). By fitting the data into a Hill equation *I*/*I*_0_=1/[1+([*C*_*Allicin*_]/*IC*_50_)nH], the concentration of the drug required for 50% blocking (IC_50_) was obtained, where *I*_0_ and *I* were the current amplitudes measured in the absence and presence of drugs, [*C*_*Allicin*_] is the drug concentration in the external solution, and nH is the Hill coefficient.

## Results

### Effect of Allicin on the *I*_*Na,L*_ of the ΔKPQ-SCN5A Mutation

**FIGURE 1:**
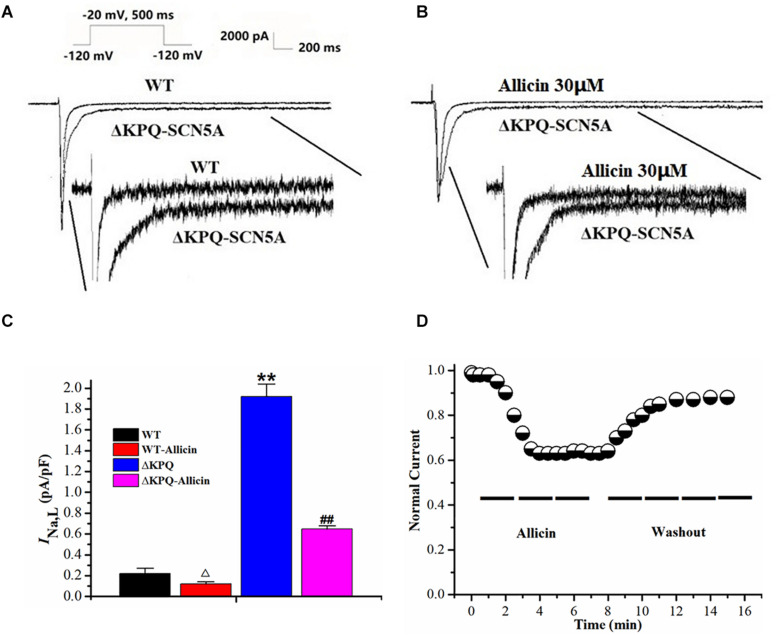
Effect of Allicin on the *I*_*Na,L*_ of the ΔKPQ-SCN5A mutation. **(A,B)** Representative current traces recorded. *I*_*Na,L*_ of ΔKPQ-SCN5A was significantly larger than that of WT, which was inhibited by Allicin. **(C)** Current densities of the *I*_*Na,L*_ in four groups were shown. *I*_*Na,L*_ of ΔKPQ-SCN5A was decreased by 30 μM Allicin from 1.92 ± 0.12 to 0.65 ± 0.03 pA/pF. **(D)** Time course of the *I*_*Na,L*_ of ΔKPQ-SCN5A inhibition by 30 μM Allicin and washout effect (*n* = 15). **P < 0.01 vs. WT group; ^##^*P* < 0.01 vs. ΔKPQ group.

*I*_*Na,L*_ densities of the ΔKPQ-SCN5A mutation were significantly higher than those of the wild-type (WT), which was increased from 0.22 ± 0.05 to 1.92 ± 0.12 pA/pF. Then, 30 μM Allicin created a significant reduction from 1.92 ± 0.12 to 0.65 ± 0.03 pA/pF (*n* = 15, *P* < 0.01) in *I*_*Na,L*_ densities of the ΔKPQ-SCN5A mutation through extracellular perfusion. Subsequently, the drug time-dependent and elution experiments were carried out. When 30 μM Allicin was added to the extracellular solution for perfusion, the current amplitude was reduced in about 1 min. The maximum inhibitory effect occurred in about 3 min, and the inhibition ratio was about 40%. After stabilization for 5 min, the current began to recover with the addition of a blank extracellular solution containing no drug, and the current density returned to 90.0% of the level before administration after elution for 5 min (Figure 1).

### Effect of Allicin on Current–Voltage and Concentration Dependence on the *I*_*Na,L*_ of the ΔKPQ-SCN5A Mutation

**FIGURE 2:**
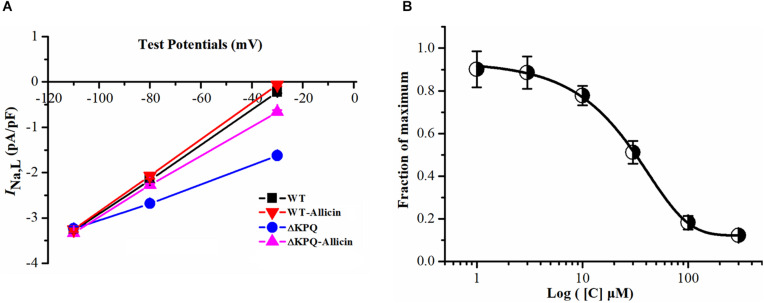
Effect of Allicin on current–voltage and concentration dependence on the *I*_*Na,L*_ of the ΔKPQ-SCN5A mutation. **(A)** Representative current–voltage relationship (voltage dependence) of the *I*_*Na,L*_ for the ΔKPQ-SCN5A mutation. **(B)** The effect of Allicin on concentration–response relationship on the *I*_*Na,L*_ of the KPQ-SCN5A mutation. IC_50_: 28.58 μM (95% CI: 20.35–39.27 μM, *n* = 15).

The current–voltage curve showed that the drug had an obvious inhibitory effect on the mutated *I*_*Na,L*_ within the stimulation range of −40 to −20 mV, which was almost close to the WT current. Allicin was added to the extracellular solution to observe the effect on the *I*_*Na,L*_ with a final concentration of 1, 3, 10, 30, 100, and 300 μM. It was found that its inhibitory effect showed a concentration-dependent characteristic with a half inhibitory concentration (IC_50_) of 28.58 μM (95% CI: 20.35–39.27 μM) and Hill coefficient of 1.13 (Figure 2).

### Effect of Allicin on the Window Current of the ΔKPQ-SCN5A Mutation

**FIGURE 3:**
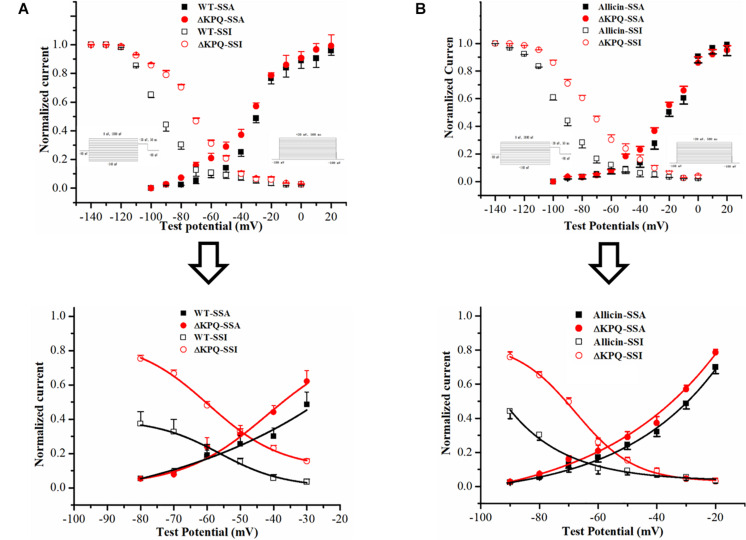
Effect of Allicin on the window current of the ΔKPQ-SCN5A mutation. **(A)** The window current of the ΔKPQ-SCN5A mutation (the area of the red cross line) is greater than that of the WT (the area of the black cross line), which indicated that the SSI curve of the WT and ΔKPQ-SCN5A mutation was different. **(B)** Under the effect of 30 μM Allicin, the SSI curve was shifted to negative potentials, and the window current of the ΔKPQ-SCN5A mutation was decreased.

As we all know, one of the main manifestations of the *I*_*Na,L*_ is the increase of the “window current,” and its magnitude is related to the SSA and steady-state deactivation of the channel (Chadda et al., 2017). Therefore, we observed the effects of Allicin on the SSA of the sodium channels. We found that under the effects of 30 μM Allicin, the SSA curve of the *I*_*Na*_ was basically unchanged. On the other hand, we discovered that the SSI curve of the ΔKPQ-SCN5A mutation was shifted to the depolarization direction and *V*_1__/__2_, _*inact*_ moved from −77.6 ± 6.1 to −65.5 ± 5.7 mV, thereby increasing the window current. After applying 30 μM Allicin, *V*_1__/__2_, _*inact*_ returned to −72.3 ± 5.7 mV (*n* = 15, *P* < 0.05), which reduced the window current (Figure 3).

### Effect of Allicin on the Dynamics of the ISI and CSI of the ΔKPQ-SCN5A Mutation Current

**FIGURE 4:**
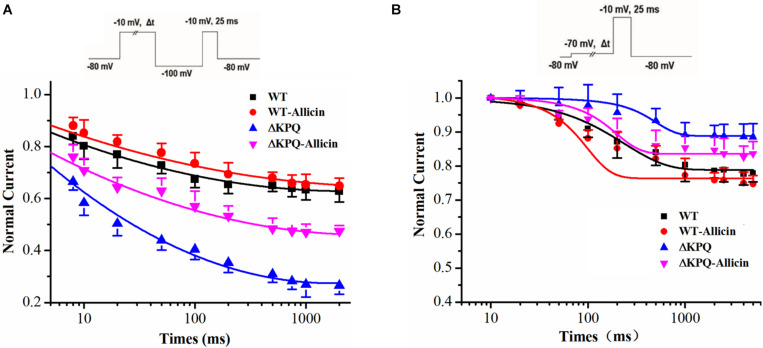
Effect of Allicin on the intermediate-state inactivation (ISI) and closed-state inactivation (CSI) of the ΔKPQ-SCN5A mutation current. **(A)** Time constant of the ISI of the ΔKPQ-SCN5A mutation current were changed by 30 μM Allicin. **(B)** Representative time constants of the CSI were not affected by 30 μM Allicin.

As shown in Figure 3, compared with the WT, the inactivation dynamics of the ΔKPQ-SCN5A mutation channel have changed. Based on the findings, we further studied the dynamics of the ISI and the CSI of the ΔKPQ-SCN5A mutation channel. Our study demonstrated that the time constants (*τ*) of the ISI of the ΔKPQ-SCN5A mutation current was significantly shortened, and that the *τ* value decreased from 605.5 ± 25.1 in WT group to 411.6 ± 32.5 ms in ΔKPQ group. Subsequently, the *τ* value of the ΔKPQ-SCN5A mutation recovered to 537.3 ± 11.4 ms under the use of Allicin (*n* = 15, *P* < 0.05). However, there is no significant difference in the time constant of the CSI in the WT, ΔKPQ-SCN5A mutation, and before and after treatment with medication (Figure 4).

### Effect of Allicin on the Nav1.5 Channel Protein Expression of the Membrane in the ΔKPQ-SCN5A Mutation

**FIGURE 5:**
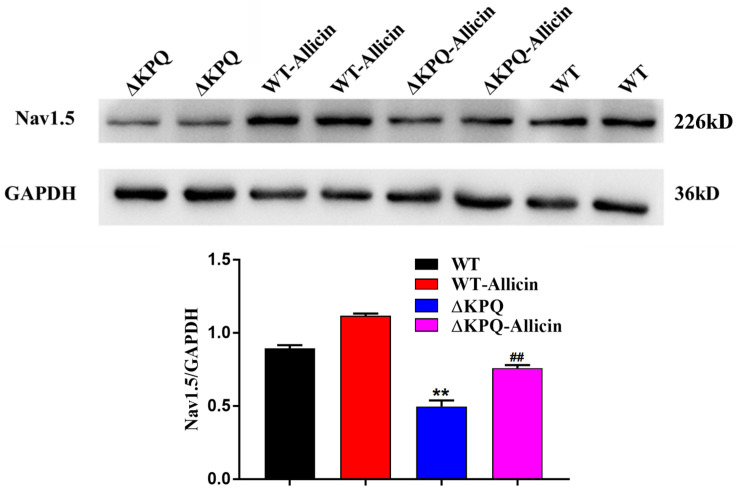
Effect of Allicin on the Nav1.5 channel protein expression of the ΔKPQ-SCN5A mutation. A Western Blotting showed that the Nav1.5 channel protein expression in cell membrane of the ΔKPQ-SCN5A mutation was increased from 0.49 ± 0.04 to 0.76 ± 0.02 under the effect of 30 μM Allicin, which was close to 0.89 ± 0.02 of the WT. ^∗∗^*P* < 0.01 vs. WT group; ^##^*P* < 0.01 vs. ΔKPQ group.

As seen in Figure 5, the Nav1.5 channel protein levels of membrane in the ΔKPQ-SCN5A mutation were enhanced from 0.49 ± 0.04 to 0.76 ± 0.02 with the effect of 30 μM Allicin, close to 0.89 ± 0.02 of the WT. It indicated that the impact of Allicin may have a defensive effect on the protein of the Nav1.5 channel or may influence the distribution and the migration of the Nav1.5 protein, thereby the amount of the membrane Nav1.5 protein expression was raised.

### Effect of Allicin on the Ratio of the *I*_*Na,L*_/*I*_*Na*,__*P*_ of the ΔKPQ-SCN5A Mutation

**FIGURE 6:**
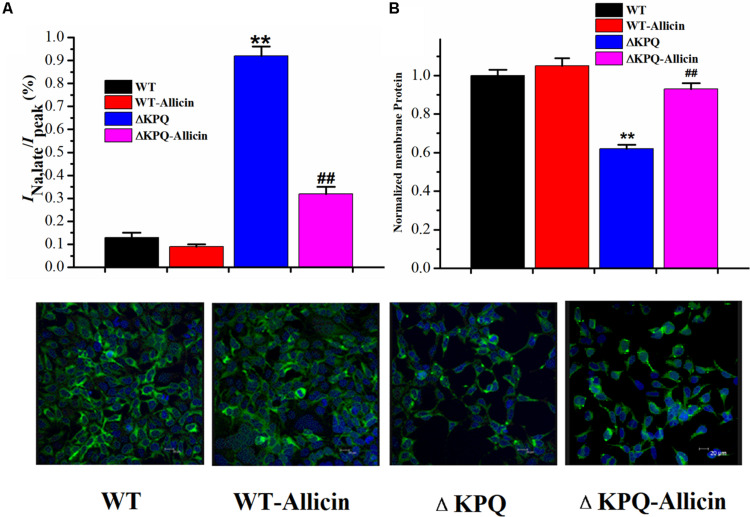
Effect of Allicin on the ratio of the *I*_*Na,L*_/*I*_*Na,P*_ and the cellular distribution of the Nav1.5 channel protein on the plasma membrane. **(A)** The incremental ratio of the *I*_*Na,L*_/*I*_*Na,P*_ of the ΔKPQ-SCN5A mutation current was decreased from 0.94% ± 0.04% to 0.32% ± 0.02% under the effect of 30 μM Allicin, which was close to the WT. **(B)** Confocal imaging of the Nav1.5 channel protein expression on HEK293 cells in the WT, WT-Allicin, ΔKPQ, and ΔKPQ-Allicin groups. ^∗∗^*P* < 0.01 vs. WT group; ^##^*P* < 0.01 vs. ΔKPQ group.

As shown in Figure 6, under the effect of 30 μM Allicin, the ratio of the *I*_*Na,L*_/*I*_*Na,P*_ of the ΔKPQ-SCN5A mutation reduced from 0.94% ± 0.04% to 0.32% ± 0.02%(*n* = 15, *P* < 0.01), which was close to the WT group. Fluorescence detection indicated that the distribution of the ΔKPQ-SCN5A mutant sodium channels in the cell membrane was significantly lower than that of the WT, which revealed the existence of channel migration defects. After co-culture of 30 μM Allicin with HEK293 carrying the Nav1.5 sodium channel encoded by ΔKPQ-SCN5A for 24 h, the distribution of the sodium channel in the cell membrane increased, which had a certain preservation effect on the mutant channel protein. It was suggested that Allicin can not only inhibit the *I*_*Na,L*_ of the ΔKPQ-SCN5A mutation but also increase the distribution of the sodium channels on the cell membrane, thus increasing the *I*_*Na,P*_ and further reducing the ratio of the *I*_*Na,L*_/*I*_*Na,P*_.

## Discussion

Wang et al. (1995) confirmed that the ΔKPQ-SCN5A mutation is one of the first SCN5A mutations to be reported in 1995. The electrophysiology of its cells was manifested by the obstacles of channel inactivation, the continuous opening of sodium channels, and the increase of late sodium current. Meanwhile, its typical clinical manifestation is prolonged QT interval and susceptible to syncope or sudden death (Li et al., 2020).

In this experiment, Allicin, the main active component, was shown to be able to efficiently decrease the late sodium current increased by the mutation of ΔKPQ-SCN5A, thus lowering the ratio of the *I*_*Na,L*_/*I*_*Na,P*_ from 0.94% ± 0.04% to 0.32% ± 0.02%. This finding provides an experimental basis for the clinical application of Allicin in treating LQT3. Class I antiarrhythmic sodium channel blockers, such as mexiletine and flecainide, have been found to effectively shorten the repolarization duration in LQT3 patients with sodium channel mutation and increased late sodium current, which is often used as gene-specific drugs in the treatment of congenital LQT3 (Benhorin et al., 2000; Windle et al., 2001; Chorin et al., 2018; Kim et al., 2019). Recent evidence shows that in patients with LQT3, not all sodium channel blockers play a therapeutic role-they could even induce new arrhythmias (Mocayar Maron et al., 2020; Schwartz et al., 2012). The strategy of finding new anti-hereditary LQT3 drugs against mutations of the SCN5A gene will therefore have good prospects.

Allicin can shift the SSI of mutant channels to the left, according to the channel gating mechanism study, indicating that the opening of the sodium channels is lower at the same depolarization potential, thereby reducing the “window current,” that is, the *I*_*Na,L*_. In the meantime, the drug can also reduce the time constant of the ISI of the ΔKPQ-SCN5A mutation channel, thus accelerating the slow inactivation process of channel which may be another reason for its reduction of the *I*_*Na,L*_. We noted that the SSA process and the time constants of the CSI were not affected by Allicin. Therefore, Allicin is considered to reduce the increase in late sodium current, mainly by improving the inactivation of mutant channels, especially the slow process of inactivation. In addition, studies have shown that the increase in late sodium current caused by sodium channel gene mutations is mostly related to the obstacles of channel protein migration from the cell to the membrane. Therefore, the ΔKPQ-SCN5A mutation may also cause the migration defect of the Nav1.5 channel protein. Subsequently, we further observed the expression of the Nav1.5 channel protein, analyzed the ratio of the *I*_*Na,L*_/*I*_*Na,P*_, and tracked the distribution of cell membrane channel proteins triggered by sodium channel gene mutation. We found that Allicin can improve the distribution of cell membrane mutant channel proteins, which could be the primary mechanism for raising peak sodium current. It is also different from other medications which at the same time decrease the peak sodium current, i.e., Brugada wave can be induced in LQT3 therapy at the same time.

There were certain limitations to this study. Only the effect of Allicin on the heterogeneously expressed late sodium current of ΔKPQ-SCN5A specifically was discussed. It is well known that a variety of ion currents make up cardiac electrical activity, so it is necessary to construct ΔKPQ-SCN5A transgenic animals to explore the changes of cardiac electrical activity and body surface ECG, particularly the impact of irregular QT prolongation, which would be more relevant for new drug research and development.

## Conclusion

Allicin has a cardiovascular protective effect, and its cardiovascular protective effect is attributed to its inhibitory function on ΔKPQ-SCN5A late sodium current. Allicin decreased the late sodium current of the ΔKPQ-SCN5A mutation, which mainly by improving the inactivation of mutant channels, especially the slow inactivation process, thus decreasing the window current. Therefore, the current study shows that Allicin should be granted experimental support for potential research and production of an alternative agent to minimize the occurrence of malignant arrhythmia in the LQT3 syndrome population. This study found the inhibitory effect of Allicin on the late sodium current of ΔKPQ-SCN5A and further explained the possible mechanism from two aspects of channel gating kinetics and channel protein distribution, offering experimental evidence for potential research and development of LQT3 therapy.

## Data Availability Statement

The original contributions presented in the study are included in the article/Supplementary Material, further inquiries can be directed to the corresponding authors.

## Author Contributions

YC, YH, and JB completed the experiments. CL, SM, JL, XL, ZF, LF, YL, and JZ contributed to the conception, drafted the manuscript, critically revised the manuscript, gave final approval, and agreed to be accountable for all aspects of work ensuing integrity and accuracy. All authors contributed to the article and approved the submitted version.

## Conflict of Interest

The authors declare that the research was conducted in the absence of any commercial or financial relationships that could be construed as a potential conflict of interest.

## Supplementary Material

Supplementary Figure 1 Steady-state activity (SSA) of *I*_*Na*_ curves.

Supplementary Figure 2 Steady-state inactivity (SSI) of *I*_*Na*_ curves.

Supplementary Figure 3 Intermediate-state inactivation (ISI) of *I*_*Na*_ curves.

Supplementary Figure 4 Closed-state inactivity (CSI) of *I*_*Na*_ curves.
