# Supplementary figures and images for: Effects of Allicin on Late Sodium Current Caused by ΔKPQ-SCN5A Mutation in HEK293 Cells
Source: Front Physiol. 2021 Mar 29;12:636485. doi: 10.3389/fphys.2021.636485 (PMC8039306; doi:10.3389/fphys.2021.636485)

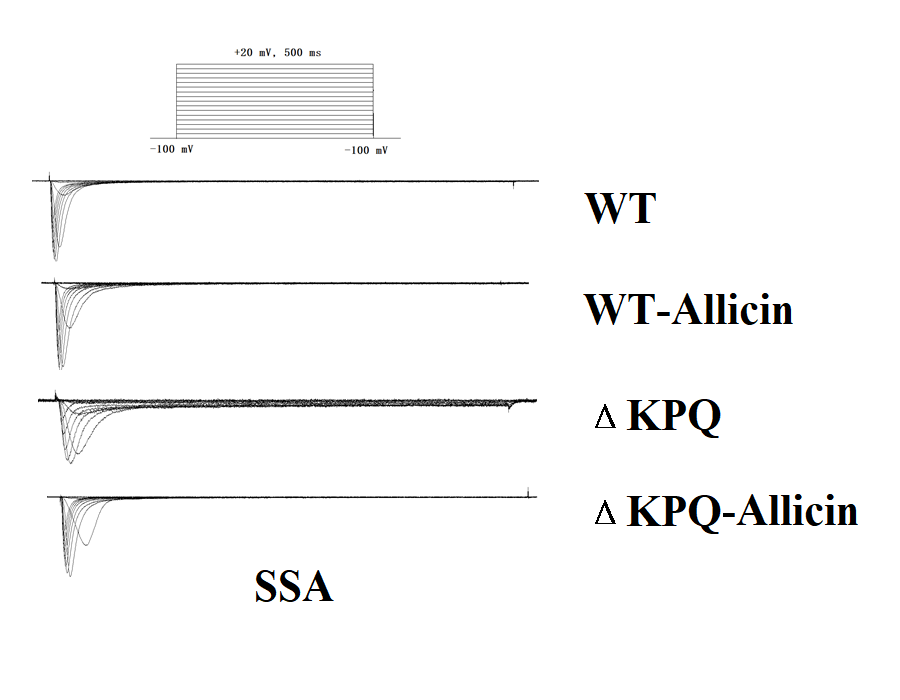

Supplement: Supplementary Figure 1 — Steady-state activity (SSA) of INa curves. [file Image_1.TIF]

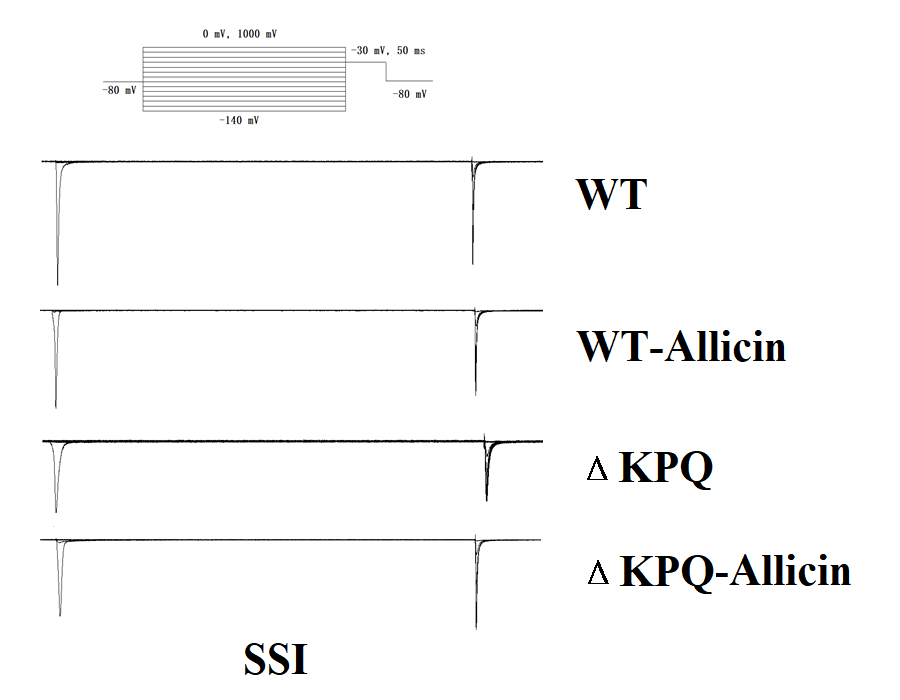

Supplement: Supplementary Figure 2 — Steady-state inactivity (SSI) of INa curves. [file Image_2.TIF]

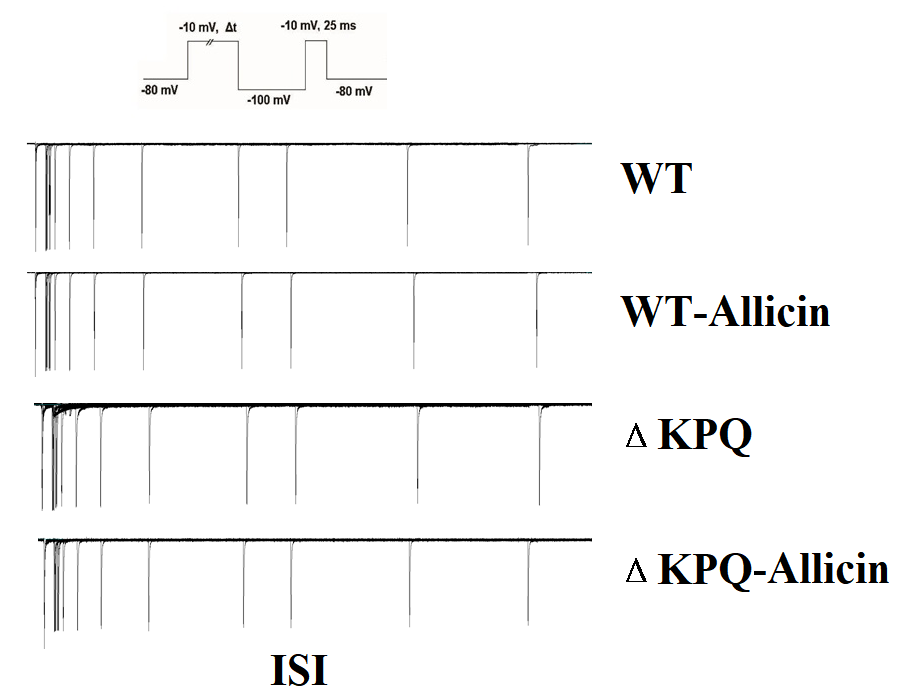

Supplement: Supplementary Figure 3 — Intermediate-state inactivation (ISI) of INa curves. [file Image_3.TIF]

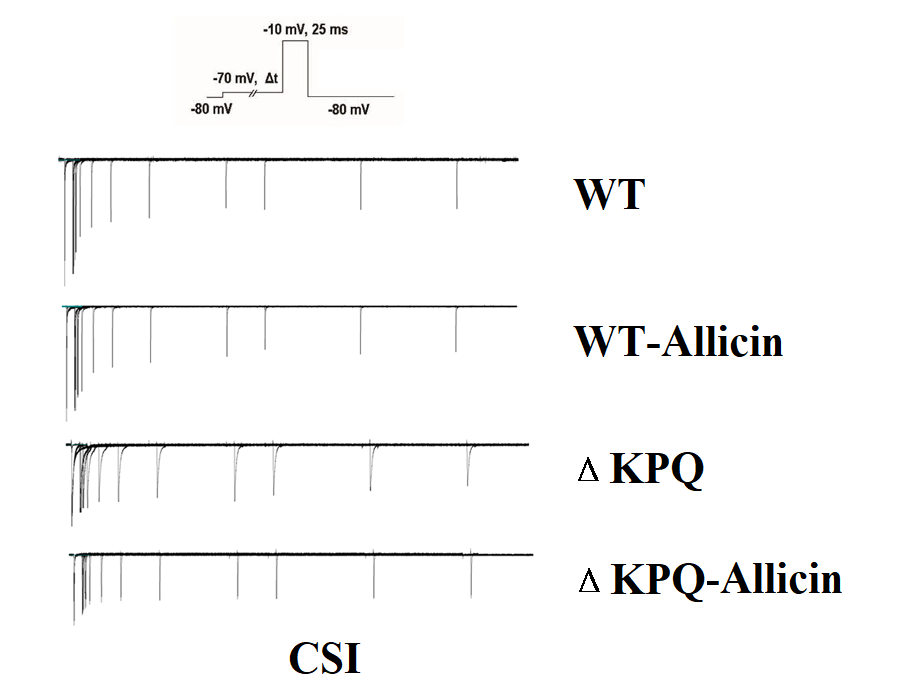

Supplement: Supplementary Figure 4 — Closed-state inactivity (CSI) of INa curves. [file Image_4.TIF]
